# Development of Key Performance Indicators for Capturing Impact of Pharmaceutical Care in Palestinian Integrative Healthcare Facilities: A Delphi Consensus Study

**DOI:** 10.1155/2020/7527543

**Published:** 2020-06-25

**Authors:** Ramzi Shawahna

**Affiliations:** ^1^Department of Physiology, Pharmacology and Toxicology, Faculty of Medicine and Health Sciences, An-Najah National University, Nablus, State of Palestine; ^2^An-Najah BioSciences Unit, Centre for Poisons Control, Chemical and Biological Analyses, An-Najah National University, Nablus, State of Palestine

## Abstract

**Background:**

The current study was performed to develop a consensus-based core inventory of key performance indicators (KPIs) to be used in capturing the impact of pharmaceutical care in healthcare facilities that employ integrative medicine paradigm in Palestine.

**Methods:**

A panel of healthcare professionals and risk/quality assurance managers was composed employing a judgmental sampling technique. The study tool was a questionnaire. Views and opinions of the panelists on the roles of pharmacists in caring for patients admitted to or visiting healthcare facilities that employ integrative medicine were collected using 11 statements. An initial inventory of activities and services that potentially can be used as KPIs was compiled from the literature and interviews with key contact experts in the domain. Three iterative Delphi rounds were conducted among the panelists (*n* = 50) to achieve formal consensus on the KPIs that should be used. The consensus-based KPIs were ordered by the scores of the panelists.

**Results:**

A total of 8 consensus-based KPIs were developed. The KPIs related to the number of problems related to medications and complementary and alternative medicine (CAM) that were resolved by pharmacists and CAM practitioners (*p* < 0.0001), number of patients for whom reconciliations were documented (*p* < 0.0001), number of patients receiving direct, comprehensive, and/or collaborative care (*p* < 0.0001), and number of patients for whom pharmacists and CAM practitioners were involved in implementing a therapeutic plan (*p* < 0.05) were rated significantly higher than the KPI (#8) related to the participation in multi-healthcare provider discussions/deliberations.

**Conclusions:**

Consensus-based KPIs that can be used in capturing the impact of evidence-based CAM and pharmaceutical care of patients in healthcare facilities that employ integrative medicine paradigm were developed. Future studies are still needed to investigate if implementing these KPIs might promote evidence-based CAM and pharmaceutical care in healthcare facilities that employ the integrative medicine paradigm.

## 1. Introduction

Improving approaches to healthcare delivery has been given a high priority across healthcare delivery systems around the globe. As a result, approaches to care are becoming increasingly person-centered and holistic in nature [1]. The concept of integrative medicine has emerged as a collaborative approach to healthcare that brings together conventional Western medicine and complementary and alternative medicine (CAM) in a coordinated way [2, 3]. Healthcare facilities employing integrative medicine cater to the needs of different patients by formulating personalized care plans that take into consideration the health needs of the body, mind, spirituality, and belonging to the community [4–6].

Studies have shown that patients are not always satisfied with the outcomes of their conventional Western medicines. Therefore, different modalities of CAM are increasingly being used by hospitalized patients as well as outpatients [7–13]. The different modalities of CAM are either used as complementary or as alternatives to conventional Western medicines [14, 15]. Although efforts are surmounting to introduce the concept of “evidence-based” to the discipline, integrative medicine approaches were shown to improve the care of patients with cancer [16], stroke [17], cardiovascular [18], inflammatory [19], and many other disease conditions [20, 21]. Studies have shown that integrative medicine reduced mortality and morbidity as well as improved patients' reported quality of life and healthcare costs. It has been argued that, in integrative medicine paradigms, healthcare providers understand the strengths and weaknesses of both conventional medicine and CAM and can blend the best parts of both approaches [2].

In modern healthcare systems, delivery of care is provided by many healthcare providers including physicians, pharmacists, nurses, and CAM practitioners. Recently, the competencies of the pharmacist as a member of the healthcare delivery team have been recognized. As a result, the roles and responsibilities of pharmacists are expanding continuously. These expanding roles and responsibilities were advocated and promoted by professional groups in different nations like the UK, USA, Australia, and Canada [22–24]. Pharmacists are experts in pharmacotherapy who are supposed to provide pharmaceutical care. Ensuring that the patients are making the best out of their therapies is one of the main roles of pharmacists. Additionally, pharmacists can provide many services that might include designing and implementing therapeutic plans, ordering laboratory tests, screening for, identifying, explaining, and resolving adverse medication reactions, interactions, ineffective therapies, and counseling/educating patients how to benefit the most out of their therapies [25–27].

In contemporary healthcare systems, there has been a shift from delivering a larger volume of care services to delivering larger quality of care services [28]. Recently, ensuring consistency of service delivery and assessing the quality of services provided has drawn considerable attention [28–31]. As a consequence, quality measures have been developed to measure and benchmark the performance of the different providers of care services across the continuum of healthcare delivery [28, 32]. To serve this purpose, key performance indicators (KPIs) are often developed for capturing the performance of healthcare providers and the provision of services. These KPIs are supposed to monitor if healthcare services were provided with consistency and efficiency. KPIs can be defined as discrete events that when they occur can result in positive outcomes for patients [28, 29, 31]. As efforts are surmounting to enhance healthcare delivery in healthcare facilities employing integrative medicine, measuring performance becomes imperative.

KPIs are increasingly used by policymakers to make decisions related to justifying the allocation of resources, improvement of quality of care, promotion of accountability, improvement of patient safety, and helping patients make informed decisions while receiving healthcare services [29]. In a recent study, 8 consensual KPIs that can be used in capturing the impact of pharmacists while providing care for patients with epilepsy in primary healthcare settings were developed [28]. In another study, Fernandes et al. developed another 8 KPIs to capture the performance of clinical pharmacists while caring for patients in hospitalized patient settings [31]. In a recent scoping review, 42 pharmaceutical activities and services were identified as potential KPIs that could be used to benchmark pharmaceutical care in integrative medicine [33]. These potential KPIs were related to activities like taking the history, reconciliation, resolving drug-related discrepancies, providing direct and collaborative care, development of care plans, improving performance, and continuing education. In another study, 9 CAM practitioners, 8 pharmacists, 2 physicians, 2 nurses, and 1 risk/quality assurance manager were interviewed in semistructured interviews to explore their perspectives on activities and services that might be used as quality indicators of pharmaceutical care in integrative medicine [34]. Thematic analysis of the interviews led to 6 major themes that were related to providing collaborative care, services at admission, during the stay, at the transition of care, and at discharge, resolving drug-related discrepancies, collaboration with other healthcare professionals, professional development, and performing services efficiently.

The literature did not narrate extensively on which activities and services that should be used as KPIs to capture the impact of pharmacists in integrative healthcare facilities. In the absence of consensus on which activities and services that should be captured while gauging the impact of pharmacists, decision-makers are left wondering on which activities and services to measure. Therefore, this study was conducted to develop and achieve consensus on what activities and services to use as KPIs to capture and measure the impact of pharmacists in integrative healthcare facilities.

## 2. Methods

**Figure 1:**
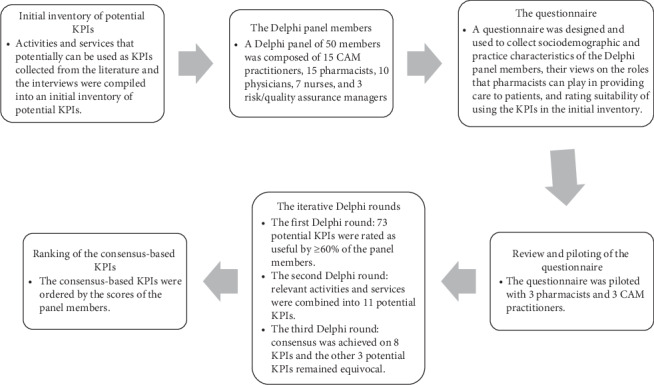
Flowchart of the different stages of the study.

This study was part of a larger study that was conducted to develop a consensus-based core inventory of KPIs to be used in capturing the impact of pharmaceutical care in healthcare facilities that employ integrative medicine paradigm [33, 34]. The present study is being reported in compliance with the Conducting and REporting of Delphi Studies (CREDES) guidelines [35]. Compliance with the CREDES is shown in Supplementary Table S1. The different stages of the study are shown in Figure 1.

### 2.1. Study Context

Patients in Palestine receive healthcare services from three main providers: (1) governmental sector, (2) private sector, and (3) international and nongovernmental organizations like the United Nations Relief and Works Agency for Palestine Refugees (UNRWA) [36]. In addition to conventional Western medicine, many Palestinians turn to the different modalities of CAM [7, 8, 10–13, 28]. These CAM modalities are either used to complement or as alternatives to conventional Wester medicines. Pharmacy schools in Palestine train pharmacists in two different academic programs: Bachelor of Science (B.Sc.) in pharmacy and Doctor of Pharmacy (Pharm.D). In addition to industry and community pharmacy practice, the pharmacy graduates are trained to assume all roles in healthcare facilities including primary, secondary, and tertiary healthcare facilities. Pharmacists are the main providers of CAM [8, 11]. As recent studies have reported a worldwide increase in using CAM modalities among hospitalized patients [14, 15], like their peers elsewhere, pharmacists in Palestine are expected to deliver a variety of pharmaceutical care services to patients using such modalities, particularly, those admitted to healthcare facilities that employ integrative medicine paradigm.

### 2.2. Study Design

This study was conducted in an observational design in which the Delphi technique was used as a formal consensus approach.

Activities performed and services provided by pharmacists while caring for patients admitted to or visiting healthcare facilities that employ integrative medicine that potentially can be used as KPIs were collected from the literature into an initial inventory [33]. The list of activities and services collected from the literature was supplemented by additional activities and services that were provided by 9 CAM practitioners, 8 pharmacists, 2 physicians, 2 nurses, and 1 risk/quality assurance manager who were interviewed using in-depth semistructured qualitative interviews [34].

### 2.3. Study Tool

The tool used in this study was a questionnaire. The questionnaire contained 3 parts. Part 1 was designed to collect sociodemographic and practice characteristics of the participants. In this part, the participants needed to provide their gender, age, profession, academic degrees, and employer. Part 2 was designed to expose the views and opinions of the participants on the roles of pharmacists in caring for patients admitted to or visiting healthcare facilities that employ integrative medicine. This part contained 11 statements on the roles that pharmacists could play in healthcare facilities that employ an integrative medicine paradigm to patient care. The participants had to express their opinions and views by selecting either disagree, neutral, or agree on each statement. Part 3 was designed to include the activities and services that were collected in the initial inventory.

#### 2.3.1. Review and Piloting the Questionnaire

The questionnaire was pilot tested with 3 CAM practitioners and 3 pharmacists for readability and comprehension (Figure 1). Based on the feedback provided by the CAM practitioners and pharmacists, wordings of some items in the questionnaire were revised for readability and clarity.

#### 2.3.2. Delphi Panel

For this study, a panel of healthcare professionals (CAM practitioners, pharmacists, physicians, nurses, and risk/quality assurance managers) was composed employing a judgmental sampling technique (Figure 1). The potential panelists were identified, invited, and recruited using personal contacts in the field. In the Delphi technique, selecting the panel members is one of the most sensitive steps [37]. Previous knowledge of the topic being investigated is a prerequisite for qualifying as a potential panel member. The panel members in this study were identified, invited, and recruited on the basis of their practical experience in the domain. The panel members were diversified by gender, age group, academic degree, profession, employer, and length of experience in the domain. The study design and objectives were explained to the potential panel members upon invitation. Before participation, all potential panel members had to provide their informed consent. In this study, the inclusion criteria were as follows: (1) having an academic degree in one of the healthcare professions with relation to integrative medicine, (2) being employed as a CAM practitioner, pharmacist, physician, nurse, or risk/quality assurance manager, (3) having practical experience in the domain for more than 5 years, and (4) willing to provide an informed consent. Potential panelists who were most likely would meet the inclusion criteria were initially invited. A total of 58 potential panel members were initially invited to this study. We intentionally invited physicians, nurses, and risk/quality assurance managers to the panel to ensure the inclusion of other members of the healthcare provision team. The number of the panel members used in this study was informed by previous studies in which the Delphi technique was used in healthcare [9, 11, 12, 28, 35, 36, 38–49]. However, there is no formal consensus on the size of panel members in the Delphi technique and panel members in previous studies ranged from 10 to 1,000 [50].

#### 2.3.3. The First Delphi Round

Questionnaires were delivered to all panel members who provided consent to participate in the study using Google Forms (Figure 1). To preserve the privacy of the panelists, the link to the questionnaire was sent to each panelist in an individualized e-mail. In the first Delphi round, the panel members provided their sociodemographic and experience characteristics, expressed their views and opinions on the 11 statements on the roles of pharmacists in providing healthcare to patients admitted to or visiting healthcare facilities that employ integrative medicine, and rated each activity and service in the initial inventory using a Likert-scale of 1–3, where scoring 1 indicated that the panel member was of the opinion that the activity or service was not useful and should not be used as a potential KPI, scoring 2 indicated that the panel member was indecisive if the service or activity was useful or not, and scoring 3 indicated that the panel member was of the opinion that the activity or service was useful and should be used as a potential KPI [28, 36].

In this round, the panel members suggested combining relevant activities together in an elaborated statement rather than using all activities and services discretely.

*(1) Data Analysis in the First Delphi Round*. Scores of the panel members were collected in an Excel Spreadsheet (Microsoft Inc.). Percentages of the panel members who rated the activity or service as useful were computed for each activity and service separately. It was decided *a priori* that activities and services rated useful by ≥60% of the panel members will be included and carried forward in the subsequent iterative Delphi rounds [51].

#### 2.3.4. The Second Delphi Round

Activities and services that were rated as useful by 60% or more of the panel members in the first Delphi round were included and carried forward to the second iterative Delphi round. Relevant activities and services were combined as was suggested by the panel members in the first Delphi round. In the second iterative Delphi round, the panel members had to use a Likert-scale of 1–9, where scoring 1 indicated complete disagreement and scoring 9 indicated complete agreement. In the Delphi technique, many indices have been previously used to express disagreement/agreement on input items [52]. In the second Delphi round, a Likert-scale of 1–9 was used to provide the panelists with a wider range to express the extent of their disagreement/agreement. Furthermore, the scale of 1–9 was the most commonly used indices in expressing the extent of disagreement/agreement by the panelists in the Delphi technique. Again, the panel members were encouraged to include written qualitative comments as justification or qualification to their scores on each activity or service.

*(1) Statistical Analysis of the Scores and Definition of Consensus Used in the Second Delphi Round*. The basic descriptive statistics like quartile 1 (Q1), quartile 2 (median), quartile 3, and interquartile range (IQR) were calculated for each activity or service separately. As the panel members provided qualitative comments also, these comments were analyzed for contents by the main investigator. The main investigator also interpreted the comments and summarized them. Interpretations and summaries were sent back to the panel members for comments, feedback, additions, deletions, or corrections.

In this study, the definition of consensus was set *a priori* and was informed by previous studies that employed the Delphi technique [9, 11, 12, 28, 36, 45–49, 51]. Results were analyzed as follows: (1) a decision was made to disregard and remove the activity or service from the final core list of KPIs when the median was in the range of 1–3 and the IQR was ≤2, (2) a decision was made to include the activity or service in the final core list of KPIs when the median was in the range of 7–9 and the IQR was ≤2, and (3) a decision could not be made whether to include or remove an activity of service from the final core list of KPIs when the median was in the range of 4–6 and/or the IQR was >2 and the decision remained equivocal. A decision was made *a priori* to carry forward and subject all equivocal activities and services in the second iterative Delphi round to a third Delphi round.

#### 2.3.5. The Third Delphi Round

Activities and services that remained equivocal in the second Delphi round were included and carried forward to the third Delphi round (Figure 1). For each equivocal activity and service, the panel members were provided with a reminder of their own scores, median scores of other panel members, and a summary of the qualitative comments made on each activity or service. The panel members were asked if they wished to maintain or reconsider their scores in view of the comments and scores of other panel members. The data were collected in the period of June to October 2019.

*(1) Analysis of Scores in the Third Delphi Round*. Scores of the panel members were analyzed using the same descriptive statistics that were used in the second Delphi round. Based on the qualitative comments provided by the panel members, it was clear that consensus was unlikely in a fourth Delphi round. Therefore, we decided not to conduct a fourth Delphi round, and the items were not included in the final core list.

*(2) Statistical Analysis of Ratings of the KPIs on Which Consensus Was Achieved*. Scores of the panel members on each KPI on which consensus was achieved were entered into GraphPad Prism for Windows (v.6.0). Dunn's multiple comparisons tests were used to compare the scores on each consensus-based KPI. Findings were considered statistically significant as follows: ^*∗*^ when *p* was <0.05, ^*∗∗*^ when *p* was <0.01, ^*∗∗∗*^ when *p* was <0.001, and ^*∗∗∗∗*^ when *p* was <0.0001.

### 2.4. Ethical Approval

This study received approval from the Institutional Review Board (IRB) of An-Najah National University. The panel members knew that the Delphi technique was semianonymous which meant that their identities were known to the investigator but not the rest of the panel members. All panel members provided informed consent before they could take part in the study.

## 3. Results

### 3.1. Panel Members

Of the 58 initially invited potential participants, 50 panelists participated in the subsequent iterative Delphi rounds (response rate = 86.2% of those initially invited). The response rate was 100% in the three iterative Delphi rounds. Of the panelists, 44% were female in gender and 78% were 40 years and older. More than half (60%) of the recruited panelists were intentionally either CAM practitioners or pharmacists. Other healthcare professionals like physicians and nurses who had different academic degrees were also represented in the panel. Half (50%) of the panelists were employed by hospitals. The majority (64%) of the panelists had practice experience of 10 and more years. Details of the sociodemographic and practice characteristics of the panelists are shown in Table 1.

### 3.2. Views and Opinions of the Panel Members on the Roles of Pharmacists in Caring for Patients Admitted to or Visiting Healthcare Facilities That Employ Integrative Medicine

When the panel members were requested to express their views and opinions on the roles that pharmacists could play in caring for patients admitted to or visiting healthcare facilities that employ integrative medicine, 94% agreed that they could play a crucial role, 86% agreed that their interventions could significantly improve care services, 90% agreed that their interventions could significantly reduce problems related to medications and CAM used by the patients, 94% agreed that they should play an active role in designing care plans, 92% agreed that they should review, evaluate, and when necessary should recommend changes to care plans, 58% agreed that currently pharmacists were underemployed in providing care, 92% agreed that their interventions might improve knowledge of patients with regard to medications and CAM, 94% agreed that they should educate patients about their diseases, medications, and CAM use, 90% agreed that they should promote adherence to medications and CAM use, 90% agreed that their interventions could promote adherence of patients to medications and CAM, and 74% agreed that their interventions could promote the quality of life of the patients. Details of the responses of the panel members are shown in Table 2.

### 3.3. Iterative Delphi Technique Rounds

#### 3.3.1. The First Delphi Round

In the first Delphi round, 73 activities and services were rated as useful by 60% and more of the panel members. These activities and services are shown in Table 3. Activities that were rated as useful by less than 60% of the panel members are shown in Supplementary Table S2.

The panel members also provided qualitative comments indicating that the inventory was in their opinion extensively huge and they suggested that relevant activities and services could be combined together. For example, a pharmacist stated: “Many of these items are related services. Allergies to drugs and CAM, drug and CAM interactions, inappropriate doses are all drug or CAM-related problems and all could be resolved by pharmacists. I think it would be better to combine them together.” Informed by similar qualitative comments of the panel members, relevant activities and services were combined into 11 items that were presented to the panel members in the second Delphi round.

#### 3.3.2. The Second Iterative Delphi Round

Relevant activities and services rated as useful by ≥60% of the panel members were combined together into 11 items and were presented to the panel members for rating and comments in the second Delphi round. The *a priori* set definition of consensus used in this study indicated that the median score had to fall within the range 7–9 and the IQR had to be ≤2 for each item to be included in the final core list of the consensus-based KPIs. As either the median fell in the range 4–6 or the IQR was larger than 2 as shown in Tables 4 and 5, items not meeting the consensus criteria were subjected to a third iterative Delphi round.

#### 3.3.3. The Third Delphi Round and the Consensus-Based KPIs

When the 11 items were presented to the panel members for scores and comments in the third Delphi round, consensus as per the definition set *a priori* was achieved on 8 (72.7%) of the 11 presented items. The consensus-based KPIs are listed in Table 4 ordered by the percentage of the panel members who rated the KPI 7–9. The 8 consensus-based KPIs were from the thematic areas of care, reconciliation, counseling/education, competence/performance/satisfaction, and multi-healthcare provider patient care.

When the scores of the panel members were compared using Dunn's multiple comparisons tests, KPIs related to the number of problems related to medications and CAM that were resolved by pharmacists and CAM practitioners (*p* < 0.0001), the number of patients who received documented reconciliation (*p* < 0.0001), the number of patients who received direct, comprehensive, and/or collaborative care (*p* < 0.0001), and the number of patients for whom pharmacists and CAM practitioners were involved in planning/preparing/implementing/executing/completing a therapeutic plan (*p* < 0.05) were rated significantly higher than the KPI (number 8) related to participation in multi-healthcare provider discussions/deliberations. The details of the multiple comparisons are shown in Supplementary Table S3.

#### 3.3.4. Activities and Services That Potentially Might Be Used as Key Performance Indicators on Which Consensus Was Not Achieved

In this study, the consensus was not achieved as per the *a priori* set definition on 3 (27.3%) items out of the 11 items presented to the panel members in the third Delphi round. Details of these items are shown in Table 5. The decision either to use these activities and services as KPIs is left to the decision and policymakers in individual healthcare facilities that employ the integrative medicine paradigm. The 3 items belonged to the thematic areas of care and professional development.

## 4. Discussion

Pharmacists are increasingly assuming roles and responsibilities in caring for patients in healthcare establishments that employ integrative medicine. Therefore, there is a pressing need to capture and measure their impact in providing healthcare services to patients who are admitted to or visiting these healthcare facilities. Traditionally, the largest volumes of healthcare services in hospitalized patient settings were provided by nurses and physicians. Therefore, traditionally, KPIs were developed to focus on activities that were performed by nurses and physicians. To effectively capture and measure the impact of pharmacists, the KPIs need to be tailored to the activities and services often provided by pharmacists [28, 30].

### 4.1. Summary and Significance of the Main Findings

In this study, consensus-based KPIs were developed for the first time to capture the impact of pharmacists in caring for patients admitted to or visiting healthcare facilities that employ integrative medicine. Prior to this study, the literature provided little guidance on what activities and services could be used as KPIs to capture and measure the impact of pharmacists in caring for patients admitted to or visiting healthcare facilities that employ integrative medicine. This study provides for the first time 8 consensus-based KPIs in five thematic areas that can be used in capturing the impact of pharmacists.

Facing severe fund cuts, top management in healthcare are always pressurized to make difficult decision to allocate scarce resources, maintain and/or extend services, and optimize healthcare delivery to patients [29, 53]. As a result, there is a surmounting need to continuously inform decision-makers, taxpayers, and funding bodies to justify allocating economic resources, secure funds, and demonstrate value in activities and services within a business plan. Therefore, the consensus-based KPIs presented in this study could be invaluable to decision-makers and stakeholders within the healthcare sector. The KPIs provided in this study could be helpful in supporting improvements in terms of quality care provided to patients admitted to or visiting healthcare facilities that employ integrative medicine. Additionally, these KPIs might promote advancing evidence-based pharmaceutical care and evidence-based CAM practice. KPIs can also be useful in delineating expectations of the top management, healthcare providers, patients, and society at large. They might also help prioritize activities and services provided by pharmacists, assist in describing the standards and quality of pharmaceutical care, and permit benchmarking of pharmaceutical care and CAM-based practice. KPIs might also promote professional accountability and transparency as well as contributions of pharmacists in improving patient reported outcomes [28, 29, 31]. It is also noteworthy mentioning that pharmacists providing care to patients admitted to or visiting healthcare facilities that employ integrative medicine can use these consensus-based KPIs to self-reflect and identify avenues for improvements in daily practice [28, 29]. Using these consensus-based KPIs to measure the impact of pharmacists and sharing the findings might be helpful in informing decision-makers to prioritize tasks, improve workflows, and avoid redundancies. Additionally, decision-makers might use these KPIs to make sure that pharmacists provide patients with the best quality of care within a given budget. Data generated from the use of these consensus-based KPIs might be presented in the meetings of quality evaluation committees, top management, fund donors, and boards of trustees.

### 4.2. Appraisal of the Method Used in This Study

Since its inception, the Delphi technique has evolved as one of the most powerful and commonly employed formal consensus approaches in developing concepts and definitions and achieving consensus on issues that lack formal consensus in healthcare. Traditionally, formal consensus techniques have been employed as alternative methods to anecdotal and subjective approaches. Advantages of the Delphi technique are numerous and include overcoming geographical barriers and constrains in reaching for panelists who possess previous knowledge of the topic being studied, eliminating financial costs needed to transport panelists and convene face to face meetings, ability to maintain the anonymity of the panel members, and ability to prevent domination of the discussion by prominent and extrovert panel members who can impose generalized bandwagon effect on the discussion [28, 43, 44].

In this study, the panel members who rated the items in the Delphi rounds again were of both genders, from different professions, had different academic degrees, were employed in different sectors, and had a comparatively long experience in the domain. The majority of the panel members were pharmacists. Other healthcare professions were also included to impart diversity to the panel and ensure the representation of other healthcare providers. This diversity might have added validity and strength to our method used in this study and to the suitability of using the developed KPIs as gold standards for developing KPIs to capture and measure the impact of pharmacists in providing care to patients admitted to or visiting healthcare facilities that employ integrative medicine do not exist. In the absence of a gold standard, formal consensus techniques provide suitable alternative methods to anecdotal and other subjective approaches [9, 11, 12, 28, 36, 45–49, 54]. It is noteworthy mentioning that professionals are more likely to use consensus-based KPIs compared to KPIs either improvised or developed using anecdotal or subjective approaches. Therefore, consensus-based methods have been promoted as approaches to reduce bias, promote transparency, and increase strength and validity to judgmental approaches while developing concepts like KPIs [28, 54].

### 4.3. Perspectives of the Panelists on the Role of Pharmacists in Integrative Healthcare Facilities

In this study, the vast majority of the panel members were of the opinion that pharmacists should play a greater role in providing care for patients admitted to or visiting healthcare facilities that employ integrative medicine. The findings of this study were consistent with those reported elsewhere and expanding the roles of pharmacists that were advocated by professional organizations in different countries around the world [14, 55–58]. As multi-healthcare provider paradigms have been promoted in modern healthcare systems, other healthcare providers like physicians expressed the desire to learn about integrative medicine and the use of CAM modalities [14, 15, 59, 60].

### 4.4. Key Performance Indicators

In this study, the consensus was achieved to consider identifying and resolving medication and CAM-related problems as a KPI to capture the impact of pharmacists in providing care to patients admitted to or visiting healthcare facilities that employ integrative medicine. The findings of this study were consistent with a previous study in which consensus was achieved to consider activities like screening for, identifying, and addressing problems related to antiepileptic drugs as a KPI to capture the impact of pharmacists in caring for patients with epilepsy visiting epilepsy clinics [28]. In an integrative medicine paradigm, pharmacists are healthcare providers with recognized expertise in pharmaco- and CAM-based therapies. Therefore, pharmacists and CAM practitioners are expected to screen for, identify, and address problems related to using medications and different CAM modalities while caring for patients. Previous studies have shown that the inclusion of pharmacists in the health provision teams reduced medication-related problems in patients admitted to hospitals, after discharge from hospitals, and in primary healthcare practice [61–65]. It was not a surprise that this KPI was rated higher than other KPIs by the panel members who participated in this study. The consensus was also achieved to services related to medication and/or CAM reconciliation as KPI to capture the impact of pharmacists in integrative medicine. The findings of this study were consistent with those previously reported on the role of pharmacists in providing care to patients [28, 29, 31]. In daily practice, pharmacists are expected to conduct medication and CAM reconciliations, take the best possible medication and CAM histories, perform reviews, and address problems and discrepancies. Medication reconciliation is now a well-recognized area in pharmaceutical care and pharmacists are increasingly expected to conduct medication reconciliations and resolve problems and discrepancies [28, 66, 67]. Additionally, pharmacists are supposed to be actively involved and use their expertise in designing care plans tailored to the individual needs of the patients [66, 67]. This might efficiently be done by collaborating with other healthcare professionals in a multi-healthcare provider model of care [28, 31]. Educating patients on their diseases, medications, and CAM modalities might need to be conducted at different stages while the patient is admitted to or visiting the healthcare facility: at admission, during their stay, and at discharge. Pharmacists are recognized experts in medications and CAM modalities. Therefore, they are in a key position to educate patients about their diseases, medications, and CAM use [68]. In modern healthcare delivery, patients are increasingly informed about their diseases and therapeutic options available to them. In clinical practice, decisions are often made jointly between healthcare providers and patients. In order to empower patients to actively be involved in making decisions, patients should be well informed about their diseases and the therapeutic options available to them. Complaints about activities and services are being used as quality indicators by different providers. In this study, the consensus was achieved to consider the number of complaints received on services delivered by pharmacists as a KPI. The findings of this study were consistent with previous studies in which complaints were used as an indicator of dissatisfaction with a service by the service user [69]. Again, the number of errors committed by pharmacists was considered a KPI in this study. The number of errors committed by pharmacists could be used to benchmark and improve the activities and services provided by pharmacists. Minimizing errors might promote safe care delivery in healthcare facilities that employ integrative medicine. The findings of this study were contradictory to those previously reported in which consensus was not achieved to consider the number of errors made by pharmacists per a predetermined time period as a KPI to measure the impact of pharmacists in caring for patients with epilepsy [28]. Active participation and contributions of pharmacists in discussions with other healthcare providers and answering their inquiries were also considered as a KPI. Previous studies have shown that ineffective communication between healthcare providers was considered an important barrier that hindered multi-healthcare provider approaches to healthcare provision [70, 71].

Although continuing educations is crucial to professional development, in this study, the consensus was not achieved to consider the number of continuing education/training sessions either attended or delivered by pharmacists as a KPI. Again, the consensus was not achieved to consider therapeutic medication or CAM monitoring and assessment of high-risk medication or CAM orders as KPIs. These activities and services remained equivocal and the decision to capture and use them in measuring the impact of pharmacists in integrative medicine is left to the decision-makers. It has been argued that KPIs should be important, relevant to patient care, and easily measurable [69]. Therefore, consensus-based KPIs could vary with regard to specialty, settings, and patient populations [28].

### 4.5. Implications on Future Practice

In Palestine, all government hospitals in the West Bank were recently connected by an electronic health information system (HIS) that was purchased by the Ministry of Health from Avicenna Health Information Medical Systems [36]. Similarly, the UNRWA developed an in-house built electronic health record system that is being implemented in more than 100 of its health centers in Jordan, Syria, Lebanon, the West Bank, and Gaza Strip [72]. The system was developed to facilitate services relevant to common illnesses, maternal and child health, noncommunicable diseases, laboratory, and pharmacy. Electronic systems are powerful tools that may permit collecting and measuring activities performed and services delivered by healthcare providers. The consensus-based KPIs developed in this study might be helpful in providing qualitative and qualitative measures of the impact of pharmacists in providing key services consistently and efficiently. Additionally, the use of these consensus KPIs might help exposing gaps that need to be addressed, attraction of funds, justifying the allocation of scarce resources, and promotion of improvements in healthcare delivery in healthcare facilities employing integrative medicine paradigms.

### 4.6. Limitations of the Study

Notwithstanding, KPIs could be associated with a number of limitations. For instance, KPIs might fail to capture the activity that they were designed to measure [73]. In this case, KPIs might underestimate the impact of healthcare providers across the continuum of healthcare delivery. KPIs could also be associated with perverse incentives and gaming the system. There could be obstacles in implementing monitoring systems that collect information on these KPIs. They could also negatively impact the morale of healthcare providers. KPIs are often dichotomous and do not necessarily provide insight into the quality of the services provided. Simply computing the KPI could impart bias and could carry an inherent risk to the quality of care provided to patients.

The findings of this study should be interpreted after carefully taking into account the following points. First, patients or their caregivers were not included in the Delphi panel members. However, the inclusion of patients or their caregivers should have permitted exposing their perspectives and expectations from their caring pharmacists. Second, community pharmacists were also not included in the Delphi panel members. In Palestine, the majority of the registered pharmacists are practicing as community pharmacists [13, 45, 47]. However, the inclusion of community pharmacists should have permitted exposing their views and opinions. Third, the Delphi technique was used as the consensus achieving technique in this study. The use of such an approach is a limitation. However, formal consensus techniques are more robust methods when the only alternatives include subjective and/or anecdotal approaches [28]. Fourth, the size of the Delphi panel was comparatively limited. However, there is no consensus on a size for the Delphi panels and previous studies have used panels with 10 to 1,000 participants [50]. The size of the panel used in this study was within the range of the panels we used before. Fifth, a purposive sampling method was used while recruiting the panelists in the Delphi technique. Nonprobability sampling techniques have long been criticized as biased. However, due to the nature and objectives of this study, the use of probability sampling approaches was not feasible. Again, having prior knowledge of the topic being investigated is a prerequisite for a participant to qualify for inclusion in a Delphi panel. Finally, all panelists were from Palestine which might limit the generalizability of the findings of this study.

## 5. Conclusions

In this study, consensus-based KPIs that can be used in capturing the impact of evidence-based CAM and pharmaceutical care of patients admitted to or visiting healthcare facilities that employ integrative medicine paradigm were developed. Future studies are still needed to investigate if implementing these consensus-based KPIs might promote evidence-based CAM and pharmaceutical care in healthcare facilities that employ an integrative medicine paradigm to healthcare.

## Figures and Tables

**Table 1:** Sociodemographic and practice characteristics of the Delphi panel members (*n* = 50).

Characteristic	*n*	%
Gender		
Male	28	56.0
Female	22	44.0
Age (years)		
<40	11	22.0
≥40	39	78.0
Profession		
CAM practitioner	15	30.0
Pharmacist	15	30.0
Physician	10	20.0
Nurse	7	14.0
Risk/quality assurance manager	3	6.0
Academic degree		
B.Sc./Pharm.D.	22	44.0
M.Sc.	11	22.0
MD	10	20.0
Ph.D.	7	14.0
Employer		
Hospital	25	50.0
Private practice	7	14.0
Pharmacy	6	12.0
Educational/training organization	9	18.0
Regulatory body/ministry	3	6.0
Length of practical experience in the domain (years)		
5–10	18	36.0
≥10	32	64.0

B.Sc.: Bachelor of Science; CAM: complementary and alternative medicine; MD: Doctor of Medicine; M.Sc.: Master of Science; Pharm.D.: Doctor of Pharmacy; Ph.D.: Doctor of Philosophy.

**Table 2:** Views and opinions of the panel members on the roles of pharmacists in caring for patients admitted to or visiting healthcare facilities that employ integrative medicine.

#	Statement	Disagree		Neutral		Agree	
		*n*	%	*n*	%	*n*	%
1	Pharmacists have the potential to play a crucial role in providing care services to patients who are admitted/visiting integrative healthcare facilities that provide CAM.	0	0.0	3	6.0	47	94.0

2	Interventions of pharmacists can bring significant improvements in care services provided to patients who are admitted/visiting integrative healthcare facilities that provide CAM.	0	0.0	7	14.0	43	86.0

3	Interventions of pharmacists can bring a significant reduction in problems related to medications and CAM prescribed and/or used by patients who are admitted/visiting integrative healthcare facilities that provide CAM.	2	4.0	3	6.0	45	90.0

4	Pharmacists should play an active role in designing the care plans for patients who are admitted/visiting integrative healthcare facilities that provide CAM.	0	0.0	3	6.0	47	94.0

5	Pharmacists should take responsibility and review, evaluate, and when necessary should recommend changes to care plans designed for patients who are admitted/visiting integrative healthcare facilities that provide CAM.	1	2.0	3	6.0	46	92.0

6	Currently, pharmacists are underemployed in providing care for patients who are admitted/visiting integrative healthcare facilities that provide CAM.	9	18.0	12	24.0	29	58.0

7	Interventions by pharmacists have the potential to improve knowledge of patients who are admitted/visiting integrative healthcare facilities that provide CAM with regard to the medications and CAM modalities prescribed to them.	1	2.0	3	6.0	46	92.0

8	Pharmacists should assume an active role in educating patients who are admitted/visiting integrative healthcare facilities that provide CAM about their diseases, medications, and CAM use.	1	2.0	2	4.0	47	94.0

9	Pharmacists should promote and ensure adherence to medication and CAM use among patients who are admitted/visiting integrative healthcare facilities that provide CAM.	1	2.0	4	8.0	45	90.0

10	Interventions of pharmacists can promote adherence of patients who are admitted/visiting integrative healthcare facilities that provide CAM to using their medications and CAM modalities.	2	4.0	3	6.0	45	90.0

11	Interventions of pharmacists can promote the quality of life of patients who are admitted/visiting integrative healthcare facilities that provide CAM.	3	6.0	10	20.0	37	74.0

CAM: complementary and alternative medicine.

**Table 3:** Activities rated by ≥ 60% of the panel members in the first Delphi round as potential key performance indicators (KPIs).

#	Activities	First Delphi round
		% of panel members who rated the activity as potential KPI
1	Number of best possible medications histories taken	88.0
2	Number of best possible CAM histories taken	84.0
3	Number of best possible medications history reviews conducted	86.0
4	Number of best possible CAM history reviews conducted	84.0
5	Number of medication reconciliations at admission conducted	76.0
6	Number of medication reconciliations at transition (between wards/services/hospitals) of care conducted	74.0
7	Number of medication reconciliations at discharge conducted	72.0
8	Number of CAM reconciliations at admission conducted	72.0
9	Number of CAM reconciliations at transition (between wards/services/hospitals) of care conducted	70.0
10	Number of CAM reconciliations at discharge conducted	74.0
11	Number of identified/resolved discrepancies/problems related to medications	80.0
12	Number of identified/resolved discrepancies/problems related to CAM	76.0
13	Number of collaborative, direct, and comprehensive patient care services provided	72.0
14	Number of therapeutic care plans developed/prepared/implemented/completed	74.0
15	Number of interprofessional discussions initiated/participated in	62.0
16	Number of suggestions accepted by other healthcare professionals like physicians	60.0
17	Number of meetings attended	60.0
18	Number of patient education sessions conducted	68.0
19	Number of formal inquiries answered	66.0
20	Number of medication orders reviewed	74.0
21	Number of CAM orders reviewed	72.0
22	Number of medication monitoring ordered/followed up/reviewed	60.0
23	Number of CAM monitoring ordered/followed up/reviewed	60.0
24	Number of contraindications related to medications identified/resolved	80.0
25	Number of contraindications related to CAM identified/resolved	82.0
26	Number of allergies to medications identified/resolved	84.0
27	Number of allergies to CAM identified/resolved	84.0
28	Number of drug-drug interactions identified/resolved	88.0
29	Number of drug-food interactions identified/resolved	78.0
30	Number of drug-CAM interactions identified/resolved	84.0
31	Number of CAM-CAM interactions identified/resolved	80.0
32	Number of CAM-food interactions identified/resolved	78.0
33	Number of inappropriate medication doses to the patient's renal function identified/resolved	64.0
34	Number of inappropriate CAM doses to the patient's renal function identified/resolved	62.0
35	Number of medication underdoses identified/resolved	62.0
36	Number of CAM underdoses identified/resolved	60.0
37	Number of medication overdoses identified/resolved	66.0
38	Number of CAM overdoses identified/resolved	64.0
39	Number of medication doses titrated to produce a desirable therapeutic effect	60.0
40	Number of CAM doses titrated to produce a desirable therapeutic effect	60.0
41	Number of adverse drug reactions identified/resolved	72.0
42	Number of adverse CAM reactions identified/resolved	70.0
43	Number of duplicate medications identified/resolved	66.0
44	Number of duplicate CAMs identified/resolved	64.0
45	Number of ineffective medications identified/resolved	62.0
46	Number of ineffective CAMs identified/resolved	62.0
47	Number of ambiguous medication orders identified/resolved	66.0
48	Number of ambiguous CAM orders identified/resolved	64.0
49	Number of misspelled medication orders identified/resolved	60.0
50	Number of misspelled CAM orders identified/resolved	60.0
51	Number of illegibly written medication orders identified/resolved	60.0
52	Number of illegibly written CAM orders identified/resolved	62.0
53	Number of missing medications (that should have been prescribed) identified/resolved	66.0
54	Number of missing CAMs (that should have been prescribed) identified/resolved	64.0
55	Number of missing medication doses identified/resolved	78.0
56	Number of missing CAM doses identified/resolved	76.0
57	Number of missing medication frequencies of administration identified/resolved	74.0
58	Number of missing CAM frequencies of administration identified/resolved	76.0
59	Number of missing medication routes of administration identified/resolved	72.0
60	Number of missing CAM routes of administration identified/resolved	76.0
61	Number of missing medication durations of therapy identified/resolved	74.0
62	Number of missing CAM durations of therapy identified/resolved	72.0
63	Number of missing recommendations to take medications in relation to meal identified/resolved	68.0
64	Number of missing recommendations to take CAM in relation to meal identified/resolved	66.0
65	Number of medication-related problems for high alert medications identified/resolved	80.0
66	Number of CAM-related problems for highly toxic CAM identified/resolved	78.0
67	Number of documented assessments of response to a therapeutic plan involving medications initiated/implemented/completed	62.0
68	Number of documented assessments of response to a therapeutic plan involving CAM initiated/implemented/completed	60.0
69	Number of complaints on the services of pharmacists and CAM practitioners received	60.0
70	Number of errors related to medications committed	66.0
71	Number of errors related to CAM committed	64.0
72	Number of continuing educational/training sessions attended	60.0
73	Number of educational/training sessions delivered	60.0

CAM: complementary and alternative medicine.

**Table 4:** The consensus-based key performance indicators.

#	KPI	Second Delphi round			Third Delphi round			Thematic area
		M	IQR	% of panel members who rated the KPI 7–9	M	IQR	% of panel members who rated the KPI 7–9	
1	Number of medication and/or CAM related problems identified and addressed/resolved by pharmacists including contraindications, inappropriate doses (over- and/or underdoses), allergies, interactions, duplications, omissions, vague/ambiguous orders, inappropriate routes of administration, inappropriate duration of therapy, and reported ineffective therapies.	8.0	3.0	60.0	9.0	1.0	90.0	Care

2	Number^*∗*^ of patients who received documented medication and/or CAM reconciliation by pharmacists including the best possible medication/CAM history/review and/or had their medication and/or CAM-related problems and discrepancies identified and addressed/resolved.	8.0	3.5	62.0	8.0	1.0	88.0	Reconciliation

3	Number^*∗*^ of patients who received direct, comprehensive, and/or collaborative care by pharmacists.	7.0	3.5	60.0	8.0	2.0	86.0	Care

4	Number^*∗*^ of patients for whom pharmacists were involved in planning/preparing/implementing/executing/completing a therapeutic plan.	6.5	3.5	58.0	7.5	2.0	84.0	Care

5	Number^*∗*^ of patients who received formal counseling/education on their diseases and/or medications/CAM by pharmacists at the time of admission, stay, transition of care, and/or discharge from the healthcare facility.	6.5	4.0	56.0	7.0	1.0	82.0	Counseling/education

6	Number of written complaints on the services delivered by pharmacists received per a predefined period of time.	6.0	4.0	52.0	7.0	1.0	80.0	Competence/performance/satisfaction

7	Number of errors committed by pharmacists per a predefined period of time.	5.5	4.0	50.0	7.0	1.0	78.0	Competence/performance/satisfaction

8	Number of multi-healthcare provider discussions/deliberations for the purpose of improving care of patients in which pharmacists actively participated and contributed including answering formal inquiries by other healthcare providers.	5.5	4.0	48.0	7.0	0.0	76.0	Multi-healthcare provider patient care

^*∗*^Or percentage out of the total number of patients who were admitted/visited/received care in the healthcare facility per a predefined period of time. CAM: complementary and alternative medicine; IQR: interquartile range; KPI: key performance indicator; M: median.

**Table 5:** The candidate activities and services that consensus was not achieved to consider them as key performance indicators.

#	KPI	Second Delphi round			Third Delphi round			Thematic area
		M	IQR	% of panel members who rated the KPI 7–9	M	IQR	% of panel members who rated the KPI 7–9	
1	Number of continuing education/training sessions attended/delivered by pharmacists per a predefined period of time.	6.0	4.5	44.0	6.0	4.0	54.0	Professional development

2	Number of therapeutic monitoring orders for medications/CAM ordered/followed up/reviewed by pharmacists per a predefined period of time.	5.5	5.0	42.0	6.0	3.5	46.0	Care

3	Number of high-risk medication/CAM regimens assessed and followed up for therapeutic response by pharmacists on defined intervals.	5.0	5.0	40.0	5.5	3.0	44.0	Care

IQR: interquartile range; KPI: key performance indicator; M: median.

## Data Availability

Data supporting the results reported in a published article can be found in the Results section and as supplementary materials with this manuscript.

## Supplementary Materials

Supplementary Materials Supplementary Table S1: adherence to Conducting and Reporting of Delphi Studies (CREDES) guidelines. Supplementary Table S2: activities rated by <60% of the panel members in the first Delphi round as potential key performance indicators (KPIs). Supplementary Table S3: multiple comparisons of ratings made by the panel members on each consensus-based key performance indicator in the third Delphi round.
