# Supplementary material for: Development of Key Performance Indicators for Capturing Impact of Pharmaceutical Care in Palestinian Integrative Healthcare Facilities: A Delphi Consensus Study
Source: Evid Based Complement Alternat Med. 2020 Jun 25;2020:7527543. doi: 10.1155/2020/7527543 (PMC7334769; doi:10.1155/2020/7527543)
Supplement: Supplementary Materials — Supplementary Table S1: adherence to Conducting and Reporting of Delphi Studies (CREDES) guidelines. Supplementary Table S2: activities rated by <60% of the panel members in the first Delphi round as potential key performance indicators (KPIs). Supplementary Table S3: multiple comparisons of ratings made by the panel members on each consensus-based key performance indicator in the third Delphi round. [file 7527543.f1.docx]

**Supplementary materials**

**Supplementary Table S1:** Adherence to Conducting and REporting of DElphi Studies (CREDES) guidelines ([Junger *et al.* 2017](#_ENREF_32))

| **#** | **Category** | **Page/line # in the manuscript** |
| --- | --- | --- |
|  | **Rationale for the choice of the Delphi technique** |  |
| 1 | Justification/rationale for Delphi | Page 19 Lines 39-42 and Page 20 Lines 1-5. |
| 2 | Purpose well defined | Page 4 Lines 16-18. |
|  | **Planning and design** |  |
| 1 | Planning and process | Page 5 Lines 22-39. |
| 2 | Selection of experts clearly justified | Page 7 Lines 6-25. |
|  | **Study conduct** |  |
| 1 | Clear description of methods | Methods section. Pages 5-9. |
| 2 | Flow chart | Figure 1 Page 6. |
| 3 | Informational input | Page 5 Lines 25-40. |
| 4 | Clear definition of consensus | Page 8 Lines 19-27. |
| 5 | Prevention of bias | Page 8 Lines 15-18. |
| 6 | Pilot test of instruments | Page 7 Lines 2-4. |
| 7 | Interpretation and processing of results | Page 8 Lines 2-5, Lines 20-28, Lines 36-39. |
| 8 | Validity | Page 8 Lines 19-27. |
|  | **Reporting** |  |
| 1 | Transparent reporting of results | Results section. Page 10 Lines 3-10. |
| 2 | Data analysis clearly justified and reported | Tables 1-5. Pages 10-17. |
| 3 | Expert panel (Member of organization, recognized authority, relevant clinical academic expertise, profession/stakeholder) | Table 1. |
| 4 | Information of rounds | Page 12 Lines 4-6, Page 15 Lines 2-28. |
| 5 | Discussion of limitations | Page 22 Lines 13-29. |
| 6 | Adequacy of conclusions | Page 22 Lines 31-35. |

**Supplementary Table S2:** Activities rated by < 60% of the panel members in the first Delphi round as potential key performance indicators (KPIs)

|  |  | **First Delphi round** |
| --- | --- | --- |
| **#** | **Activities** | **% of panel members who rated the activity as potential KPI** |
| 1 | Number of medications reviewed in each medication history taken | 52.0 |
| 2 | Number of CAM modalities in each CAM history taken | 56.0 |
| 3 | Number of medications in each medications history review conducted | 48.0 |
| 4 | Number of CAM modalities in each CAM history review conducted | 50.0 |
| 5 | Number of medications in each reconciliation session conducted at admission | 54.0 |
| 6 | Number of medications in each reconciliation session conducted at transition (between wards/services/hospitals) of care | 48.0 |
| 7 | Number of medications in each reconciliation session conducted at discharge | 50.0 |
| 8 | Number of CAM modalities in each reconciliation session conducted at admission | 48.0 |
| 9 | Number of CAM modalities in each reconciliation session conducted at transition (between wards/services/hospitals) of care | 52.0 |
| 10 | Number of CAM modalities at each reconciliation session conducted at discharge | 46.0 |
| 11 | Number of suggestions provided to other healthcare professionals like physicians | 40.0 |
| 12 | Duration of meetings attended | 36.0 |
| 13 | Duration of the patient education sessions conducted | 42.0 |
| 14 | Duration of continuing educational/training sessions attended | 40.0 |
| 15 | Duration of educational/training sessions delivered | 38.0 |

CAM: complementary and alternative medicine, KPI: key performance indicator

**Supplementary Table S3:** Multiple comparisons of ratings made by the panel members on each consensus-based key performance indicator in the third Delphi round

| **Number of the KPI** | 1 (# of problems solved) | 2 (# of reconciliations) | 3 (# patients who received direct care) | 4 (# of therapeutic plans) | 5 (# of patients counselled) | 6 (# of complaints) | 7 (# of errors) | 8 (# of discussions) |
| --- | --- | --- | --- | --- | --- | --- | --- | --- |
| 1 (# of problems solved) | - | ns | ns | * | **** | **** | **** | **** |
| 2 (# of reconciliations) | ns | - | ns | ns | **** | **** | **** | **** |
| 3 (# patients who received direct care) | ns | ns | - | ns | * | ** | *** | **** |
| 4 (# of therapeutic plans) | * | ns | ns | - | ns | ns | ns | * |
| 5 (# of patients counselled) | **** | **** | * | ns | - | ns | ns | ns |
| 6 (# of complaints) | **** | **** | ** | ns | ns | - | ns | ns |
| 7 (# of errors) | **** | **** | *** | ns | ns | ns | - | ns |
| 8 (# of discussions) | **** | **** | **** | * | ns | ns | ns | - |

1 (# of problems solved): Number of medication and/or CAM related problems identified and addressed/resolved by CAM practitioners and pharmacists including contraindications, inappropriate doses (over- and/or under-doses), allergies, interactions, duplications, omissions, vague/ambiguous orders, inappropriate routes of administration, inappropriate duration of therapy, and reported ineffective therapies, 2 (# of reconciliations): Number* of patients who received documented medication and/or CAM reconciliation by CAM practitioners and pharmacists including best possible medication/CAM history/review and/or had their medication and/or CAM related problems and discrepancies identified and addressed/resolved, 3 (# patients who received direct care): Number* of patients who received direct, comprehensive, and/or collaborative care by CAM practitioners and pharmacists, 4 (# of therapeutic plans): Number* of patients for whom CAM practitioners and pharmacists were involved in planning/preparing/implementing/executing/completing a therapeutic plan, 5 (# of patients counselled): Number* of patients who received formal counseling/education on their diseases and/or medications/CAM by CAM practitioners and pharmacists at the time of admission, stay, transition of care, and/or discharge from the healthcare facility, 6 (# of complaints): Number of written complaints on the services delivered by CAM practitioners and pharmacists received per a pre-defined period of time, 7 (# of errors): Number of errors committed by CAM practitioners and pharmacists per a pre-defined period of time, 8 (# of discussions): Number of multi-healthcare provider discussions/deliberations for the purpose of improving care of patients in which CAM practitioners and pharmacists actively participated and contributed including answering formal inquiries by other healthcare providers.

*or percentage out of total number of patients who were admitted/visited/received care in the healthcare facility per a pre-defined period of time

Comparisons were performed using Dunn's multiple comparisons test, ns: not significant, *: *p*-value < 0.05, **: *p*-value < 0.01, ***: *p*-value < 0.001, ****: *p*-value < 0.0001

KPI: key performance indicator, CAM: complementary and alternative medicine.
